# A 3D ColMA-Based Tenogenic Microenvironment Unveils the Behavior of Tendon Stem/Progenitor Cells (TSPCs) from Tendinopathic Surgical Explants

**DOI:** 10.3390/bioengineering12121337

**Published:** 2025-12-08

**Authors:** Giacomo Cortella, Erwin Pavel Lamparelli, Joseph Lovecchio, Emanuele Giordano, Nicola Maffulli, Giovanna Della Porta

**Affiliations:** 1 Department of Medicine, Surgery and Dentistry, University of Salerno, Via S. Allende, 84081 Baronissi, SA, Italy; gcortella@unisa.it (G.C.); elamparelli@unisa.it (E.P.L.); 2 Faculty of Engineering, University Campus Bio-Medico of Rome, Via Alvaro del Portillo 21, 00128 Rome, Italy; 3 Laboratory of Cellular and Molecular Engineering “Silvio Cavalcanti”, Department of Electrical, Electronic and Information Engineering “Guglielmo Marconi” (DEI), University of Bologna, 47522 Cesena, FC, Italy; 4 Advanced Research Center on Electronic Systems (ARCES), University of Bologna, 40126 Bologna, BO, Italy; 5 Department of Trauma and Orthopaedics, Faculty of Medicine and Psychology, Sant’Andrea Hospital, Sapienza University, 00189 Rome, Italy; 6 Research Centre for Biomaterials BIONAM, Università di Salerno, Via Giovanni Paolo II, 84084 Fisciano, SA, Italy

**Keywords:** tendon stem/progenitor cells (hTSPCs), 3D bioprinting, GDF-5-loaded PLGA nanoparticles, perfusion bioreactor, tendinopathy modeling

## Abstract

Tendon injuries present significant clinical challenges due to limited intrinsic healing and complex pathological mechanisms. Here, we developed a novel 3D bioprinted methacrylated type I collagen (ColMA) scaffold integrated with Growth Differentiation Factor-5 (GDF-5)-loaded Poly (lactic-co-glycolic acid) (PLGA) nanoparticles and dynamically cultured it under perfusion to establish a tenogenic microenvironment in vitro. Pathological human Tendon Stem/Progenitor Cells (hTSPCs) derived from tendinopathic surgical explants were encapsulated to investigate their impaired extracellular matrix (ECM) deposition and associated pro-inflammatory signaling. GDF-5-loaded nanoparticles (average diameter 140 ± 40 nm) were fabricated via microfluidic-assisted nanoprecipitation and homogeneously incorporated within the ColMA synthetic ECM to enable sustained growth factor release. Continuous perfusion culture (1 mL/min) ensured efficient mass transfer and supported cell viability above 70% over 21 days. Pathological hTSPCs exhibited impaired ECM remodeling, characterized by the absence of type I collagen and a 2.56-fold increase in type III collagen at day 7, indicative of a fibrotic-like phenotype. Western blot densitometry demonstrated a 5.31-fold elevation in secreted tenomodulin at day 14, while ECM analysis verified a type III to type I collagen ratio of 4.5. In addition, a markedly pro-inflammatory cytokine profile was observed, with elevated secretion of interleukin-6 (IL-6) and interleukin-8 (IL-8) from day 7 onward, consistent with the chronic inflammatory status of cells derived from pathological tendon tissues. This modular 3D platform represents a robust in vitro model for mechanistic studies and the advancement of personalized regenerative strategies targeting chronic tendon disorders.

## 1. Introduction

Tendon injuries represent a major clinical challenge in orthopedics due to their limited healing capacity as well as unknown etiology [1]. However, tissue-engineered in vitro models have emerged as promising approaches to study the pathological environment by providing bioengineered scaffolds that support cell viability, extracellular matrix (ECM) deposition, and cell differentiation [2,3]. In this context, three-dimensional (3D) bioprinting has become a powerful tool for biofabricating biomimetic constructs that closely resemble the native tissue microenvironment to study the cellular function and behaviors [4,5]. Among the various biomaterials investigated, methacrylated collagen hydrogels have gained increasing attention to support cell proliferation and differentiation [6].

While bioinks play a fundamental role in the fabrication of three-dimensional in vitro models, the effective modulation of cellular differentiation often requires additional biochemical cues, such as growth factors, as well as tailored material properties. In this context, particular attention has been given to conductive and bioactive materials capable of supporting and directing specific lineage commitment [7,8,9]. Tenogenic differentiation, for example, is well-promoted in the presence of Growth Differentiation Factor-5 (GDF-5), a member of the Bone Morphogenetic Protein (BMP) family, at concentrations around 100 ng/mL [10,11]. Yet, direct supplementation of culture media with GDF-5 remains challenging, due to its rapid degradation and short half-life, which reduce its overall biological effectiveness [12]. Concurrently, recent advances in hydrogel-based scaffolds, especially those combining natural and synthetic polymers, have enabled the recreation of tendon-like biomechanical characteristics while providing a supportive microenvironment for cell viability and differentiation. For instance, fiber-reinforced hydrogels have been shown to improve the mechanical performance and enhance tenogenic maturation [13]. In addition, further research has focused on developing cationic hydrogel systems, which are highly desirable for tendon tissue-engineering strategies [14].

On the other hand, biodegradable Poly (Lactic-co-Glycolic Acid) (PLGA) nanoparticles (NPs) have been largely employed as a controlled-release system, ensuring sustained and localized growth factor delivery [15]. Among the various technologies for PLGA-NP formulation, microfluidics-assisted nanoprecipitation has emerged as a powerful approach, offering superior control over the NP size, uniformity, and acceptable loading [16,17]. Growth factor-loaded NPs can be integrated within a biomimetic matrix to create a microenvironment that closely resembles the ECM. Despite their ability to efficiently release specific growth factors, the bio-plotting of these NPs, for tendon tissue-engineering purposes, using 3D printers, has rarely been documented [18,19].

Human Tendon Stem/Progenitor Cells (hTSPCs) have been extensively recognized for their multipotent capabilities, enabling differentiation into various cell types, including osteoblasts, chondrocytes, and adipocytes [20]. Additionally, hTSPCs exhibit a robust self-renewal capacity, a defining characteristic of stem cells, and express key surface markers such as Cluster of Differentiation 44 (CD44), CD90, and CD105, which are essential for their identification and function. Their role in tendon homeostasis and repair is particularly significant, as they contribute to ECM remodeling and respond dynamically to mechanical and biochemical cues within the tendon microenvironment [21].

Given these properties, pathological hTSPCs derived from tendinopathic explants of patients’ tendons were recently investigated by Ciardulli et al. [22], and it was demonstrated that their expanded monolayer culture exhibited marked alterations in ECM gene expression, notably an increased COL3A1/COL1A1 ratio, persistent secretion of inflammatory cytokines, and impaired acquisition of a mature tenogenic phenotype relative to the healthy controls. These findings highlighted intrinsic disease-associated deficits in stem cell function from pathological tissue but underscored the limitations of conventional culture platforms in recapitulating the ECM deposition. In this scenario, an ECM-like 3D environment holds promise for innovative bioengineering approaches, such as integration into a biomimetic in vitro model for the investigation of the mechanisms behind tendon pathology and musculoskeletal disorders [23].

3D culture requires perfusion, which optimizes the exchange of functional biomolecules and helps maintain a more physiologically relevant microenvironment. It enables continuous nutrient delivery and efficient waste removal, both of which are essential for sustaining cell viability and function over extended culture periods. On the other hand, the 3D culture environment exploration by Finite Element Method (FEM) simulations has demonstrated the importance of controlled fluid flow in maintaining a stable and reproducible environment [24]. These simulations highlight how perfusion enhances mass transport, reduces concentration gradients, and prevents the formation of necrotic zones within dense tissue constructs. By mimicking in vivo-like conditions, perfusion promotes cell proliferation, differentiation, and ECM deposition, all of which are crucial for tissue-engineering applications. Furthermore, perfusion systems can be tailored to provide gradient concentrations that may influence cell behavior [25].

In the present study, a 3D bioplotted tendon construct was developed by incorporating pathological hTSPCs within a PhotoCol^®^ scaffold that was functionalized with PLGA-NPs loaded with GDF-5. The experimental platform was specifically designed to assess the ECM remodeling capabilities of tendinopathic cell sources. NPs were fabricated by microfluidic technology and lyophilized for further use. Cellular behavior within the bioplotted 3D environment was assessed using quantitative Real-Time Polymerase Chain Reaction (qRT-PCR) and Western blot (WB) assay to evaluate the gene expression of key tenogenic genes and ECM proteins secretion, respectively, offering critical insights into the differentiation potential and efficacy of the engineered culture. In addition to protein evaluation, histological evaluations were performed to validate both the molecular and structural outcomes of the biofabricated constructs. Furthermore, Picrosirius Red staining was employed to detect type I and type III collagen and to confirm their localization in the synthetic matrix [26]. Finally, cytokine secretion was monitored during the entire duration of the dynamic culture to deeply understand the paracrine effect in a pathological cell environment.

## 2. Materials and Methods

### 2.1. hTSPCs’ Isolation and In Vitro Expansion

Human Tendon Stem/Progenitor Cells (hTSPCs) from two tendinopathic tendons patients (TMA samples) (average age: 43 years old) were explanted after informed consent, according to protocols approved by the Institutional Review Board of “San Giovanni di Dio e Ruggi D’Aragona Hospital” (Salerno, Italy) (Review Board prot./SCCE n. 151 achieved on 29 October 2020). The extracted cells were cultured in α-Minimum Essential Medium (α-MEM, Corning) supplemented with 1% Glutagro™ (Corning), 20% fetal bovine serum (FBS, Gibco™, Waltham, MA, USA), 1% penicillin/streptomycin (Corning, Manassas, VA, USA), and 1% amphotericin B (Corning) and maintained at 37 °C in a humidified atmosphere with 5% CO_2_. Cells were expanded until passage 3 before further experimentation; further data on hTSPCs are published elsewhere [22].

### 2.2. Preparation of GDF-5-Loaded PLGA Nanoparticles

Nanoparticles (NPs) were synthesized using a microfluidic system (NanoGenerator Flex M, Precigenome LLC, San Jose, CA, USA) equipped with a Y-shaped chip featuring a staggered herringbone mixer. The microchannel dimensions were 300 µm (inlet width), 600 µm (outlet width), 200 µm (depth), and 20 mm (total length).

Poly (lactic-co-glycolic acid) (PLGA, 50:50; Resomer^®^ RG 502H, Evonik, Essen, Germany; 145 Mw 7000–17,000) was solubilized in acetonitrile at concentration of 10 mg/mL. Growth Differentiation Factor-5 (GDF-5, Peprotech, 120-01) was dissolved at 5.33 μg/mL in sterile Milli-Q^®^ water containing 1% *w*/*w* poly (vinyl alcohol) (PVA; Mol wt 30,000–70,000, Aldrich Chemical Co., Milan, Italy). An oil-to-water ratio of 1:3 and a total flow rate (TFR) of 10 mL/min were adopted during microfluidic processing. The resulting NP suspension was collected, dialyzed to remove ethanol residues, and subsequently characterized for size, polydispersity index (PDI), and ζ-potential using dynamic light scattering (DLS) and nanoparticle tracking analysis (NTA).

### 2.3. PLGA-Nanoparticles Physicochemical Characterization

The hydrodynamic diameter, Polydispersity Index (PDI), and ζ-potential were measured at 25 °C by Dynamic Light Scattering (DLS, ZetaSizer Mod. 1000HSa, Malvern, UK). The NP size distribution and concentration were further evaluated using a NanoSight NS500 (Malvern Panalytical, Malvern, UK) equipped with an sCMOS camera. Samples were diluted 1:1000 (*v*/*v*) in 0.22 μm-filtered Milli-Q water to obtain 20–200 particles per frame. Five consecutive 1 min measurements were recorded at 25 °C with a pump flow rate of 30 μL/min. The detection threshold allowed up to 10% indistinct particles, with no more than five blue-cross identifications per frame. Videos were analyzed using NanoSight NTA 3.1 Build 3.1.46 at a sensitivity setting of 5.

A Field Emission Scanning Electron Microscope (FE-SEM, LEO 1525, Carl Zeiss SMT AG, Oberkochen, Germany) was used for morphological characterization. NPs were dried in air and mounted on aluminum stubs using double-sided carbon tape and coated with a 250 Å gold layer using a sputter coater (108A sputter coater, Agar Scientific, Stansted, UK) prior to imaging. The 3D scaffold was first passed through an ethanol dehydration series and subsequently freeze-dried to preserve the particle structure.

The loading efficiency was determined by dissolving 1 mg of NPs in acetone, followed by peptide extraction via liquid–liquid extraction and its quantitative assays with an ELISA Kit (HUFI00719; DBA Italia S.r.l., Milan, Italy), with procedures strictly adhering to the kit manual to minimize the variability and ensure reproducibility. The loading was calculated adopting the following Formula (1):

(1) $$EE\left(\%\right)=\frac{W_{t}}{W_{i}}\times 100,$$

where W_t_ is the total amount of encapsulated material, and W_i_ is the total amount of material initially added during the preparation. To ensure reproducibility, three independent measurements were conducted for each batch immediately after preparation.

### 2.4. 3D Bioprinting of PhotoCol^®^-Based Constructs

A commercially available methacrylated type I collagen hydrogel (PhotoCol^®^, Advanced BioMatrix, Carlsbad, CA, USA) was used as the bioink. Briefly, the final hydrogel master mix was functionalized with a free radical initiator: LAP (Lithium phenyl-2,4,6-trimethylbenzoylphosphinate); the hTSPCs final concentration was 1.5 million cells/mL, and GDF-5 was 11.5 mg/mL. Prior to incorporating the cells and NPs, the solution was neutralized and kept chilled.

Bioprinting was performed using the Rokit INVIVO 4D2 bioprinter (ROKIT Healthcare Co., Ltd., Seoul, Republic of Korea), an extrusion-based 3D bioprinter equipped with a temperature-controlled print head that can provide a sterile environment, thanks to the incorporation of HEPA filters inside the printing chamber. Using NewCreatorK software (RokitHealthcare, Seoul, Republic of Korea, version 1.57.70), a concentric cylindrical design measuring 5 mm in diameter and 2 mm in height was created, sliced, and uploaded to the bioprinter. The printing bed temperature was maintained at 40 °C, while the extrusion chamber was kept at 18 °C. The extruder operated at a printing speed of 5 mm/s, with a travel speed of 7 mm/s. The scaffolds were printed directly onto microscope glass coverslips and crosslinked using UV irradiation at 405 nm to induce hydrogel polymerization, incubated at 37 °C and 5% CO_2_ for 15 min, and subsequently transferred to culture plates with fresh medium. Following fabrication and post-processing curing, the scaffolds were transferred to the perfusion bioreactor and cultured for up to 21 days.

### 2.5. Rheological Assessment of the Bioink and of the Bioplotted Construct

Rheological properties of the bioink were assessed at a concentration of 8 mg/mL to evaluate its viscosity. Measurements were performed with a Kinexus rheometer (Malvern Panalytical Ltd., Malvern, UK) utilizing a flow curve test, in which the viscosity was determined as a function of the shear rate in the range of 0.01 to 100 s^−1^ at a controlled temperature. Steady-state flow tests were conducted to characterize the shear-thinning behavior of the bioink, which is relevant to its printability and ability to form stable scaffolds during the bioprinting process.

After crosslinking, the viscoelastic properties of ColMA hydrogels were measured by small-amplitude oscillatory shear in frequency sweep mode at 20 °C. A constant oscillatory shear strain of approximately 2% (within the linear viscoelastic regime) was applied, while the angular frequency was swept from 0.01 to 100 Hz, with the measuring gap adjusted between 0.72 and 1.44 mm according to the sample thickness and formulation. For all samples, the storage modulus G′ and loss modulus G″ were recorded as a function of the frequency to quantify the elastic and viscous contributions of the crosslinked ColMA-based scaffolds.

### 2.6. Cell Dynamic Culture

A custom-designed perfusion bioreactor, made of Poly (Methyl MethAcrylate) (PMMA, Altuglas^®^ CN, La Garennecolombes, France) was utilized in this study [27]. The bioreactor featured a multi-well plate equipped with two apertures for the insertion of silicon tubing (Tygon^®^, Charny, France), enabling continuous medium circulation via peristaltic pumps set to a constant flow rate of 1 mL/min. This steady flow was maintained throughout the duration of the experiment. The medium was recirculated through the peristaltic system and replenished with fresh media once per week. The entire bioreactor setup was housed within a standard cell culture incubator maintained at 37 °C with 5% CO_2_.

### 2.7. Cell Viability

Cell viability was assessed using a live/dead assay on days 0, 7, 14, and 21. Scaffolds were stained according to the manufacturer’s protocol. Briefly, scaffolds were washed three times with PBS for 5 min and then incubated in a PBS solution with a solution of 2% of Calcein AM (Cat. no. C1359, Sigma Aldrich, Milan, Italy) and 1% Ethidium Homodimer-1 (Cat. no. E1903, Sigma Aldrich, Milan, Italy), for 15 min at 37 °C in the dark. After incubation, scaffolds were washed with PBS for 5 min and visualized under a fluorescence microscope (mod. Eclipse, Nikon Corporation, Tokyo, Japan) using a 488 nm excitation filter and a 525 nm emission filter. Live cells, stained green by Calcein AM, were distinguished from dead cells, stained red by Ethidium Homodimer-1. Images were captured at magnifications of 4× and 10×. The signal intensity was quantified using ImageJ software (version 1.52p, National Institutes of Health, Bethesda, MD, USA). The original images, initially in RGB format, were converted to 8-bit grayscale images. The designated regions were analyzed and reported as the mean pixel intensity values, ranging from 0 (black) to 255 (white) [28].

### 2.8. Tenogenic Differentiation and ECM Analysis

Total mRNA was isolated from scaffold samples at each experimental time point using QIAzol^®^ Lysis Reagent in combination with the RNeasy Mini Kit (Qiagen, Hilden, Germany). One microgram of purified RNA per sample was subsequently used for first-strand cDNA synthesis employing the iScript™ cDNA synthesis kit (Bio-Rad, Milan, Italy). Quantitative Real-Time PCR analysis was performed on a LightCycler^®^ 480 Instrument (Roche, Monza, Italy) utilizing the SsoAdvanced™ Universal SYBR^®^ Green Supermix (Bio-Rad, Foster City, CA, USA). Validated primer pairs targeting Scleraxis (SCX-A), Tenascin C (TNC), Collagen type I alpha 1 chain (COL1A1), Collagen type III alpha 1 chain (COL3A1), and Tenomodulin (TNMD) (Bio-Rad), were employed for gene-specific amplification according to the Minimum Information for Publication of Quantitative Real-Time PCR Experiments (MIQE) guidelines. Expression data were normalized to the reference gene GlycerAldehyde-3-Phosphate DeHydrogenase (GAPDH). Reference gene stability across experimental conditions was evaluated using the geNorm algorithm (CFX Manager v.3.1; Bio-Rad, Milan, Italy), with a threshold of M < 0.5 indicating adequate stability. Relative gene expression changes were calculated using the 2^−ΔΔCt^ method and are presented as fold-change values compared to the expression baseline measured immediately after bioprinting (Day 0). All procedures were performed in triplicate biological samples (n = 3).

Sirius Red staining was performed using the Picrosirius Red Stain Kit (Polysciences, Inc., Warrington, PA, USA). Tissue sections, each 15 μm thick, were first stained with Hematoxylin for 8 min and rinsed with water for 2 min. The sections were then treated with Phosphomolybdic Acid for 2 min, followed by another 2 min water wash. Afterward, the samples were incubated in Picrosirius Red F3BA stain for 60 min and briefly immersed in 0.1 M HCl for 2 min. The staining protocol was completed by dehydrating the sections through graded ethanol solutions (70%, 75%, 95%, and 100%), followed by a 5 min immersion in xylene. The samples were then mounted using Eukitt medium for further examination. Brightfield and polarized light images of the Picrosirius Red-stained sections were acquired at 5×, 10×, and 20× magnifications using a LEICA DM6 M microscope (Leica Microsystems, Milan, Italy). To provide an objective assessment complementing the qualitative observations from the microscope images, raw birefringence data were analyzed at days 0, 7, 14, and 21 using the Picrosirius Red Birefringence Analyzer plugin (CMM_PR-BRF_Analyser.ijm) in ImageJ/FIJI, which computes per-replicate area fractions. This quantification elucidates dynamic ECM remodeling in the 3D ColMA scaffolds seeded with pathological human tendon stem/progenitor cells (hTSPCs).

### 2.9. Protein Precipitation and Western Blot Assay

Western blot analysis was conducted to investigate the activation of signaling pathways such as Tnmd, Col1A1, and Col1A3 in response to GDF-5 stimulation. At each time point, scaffolds underwent a 15 min wash in PBS before being transferred to Buffer RLT (RNeasy^®^ Micro Kit, QIAGEN, Germany) for total protein extraction. An equal volume of 70% ethanol was added to the lysate, followed by immediate centrifugation at 8000× *g* for 15 s. Next, four volumes of ice-cold acetone were mixed in, and the samples were incubated at −20 °C for 30 min. The mixtures were then centrifuged at maximum speed for 10 min at 4 °C, and the resulting pellets were air-dried. The dried samples were resuspended in RIPA buffer (150 mM NaCl, 1% Triton X-100, pH 8.0, 0.5% sodium deoxycholate, 0.1% SDS, and 50 mM Tris, pH 8.0) supplemented with protease and phosphatase inhibitors (Merck, Rahway, NJ, USA). Protein concentrations were quantified using the Pierce™ BCA Protein Assay Kits (Thermo Scientific, Rockford, IL, USA). For analysis, 19 μg of total protein was separated by SDS-PAGE and transferred to nitrocellulose membranes.

The membranes were blocked in TBS-T buffer (20 mM Tris–HCl, pH 7.4, 500 mM NaCl, 0.1% Tween-20) containing 10% non-fat dry milk and then incubated overnight at 4 °C in TBS-T with 5% nonfat dry milk and the following primary antibodies: anti-tenomodulin (ab203676, Abcam, Cambridge, MA, USA) and anti-collagen type I (ab138492, Abcam, Cambridge, MA, USA). Immunoreactivity was visualized by sequential incubation with appropriate horseradish peroxidase-conjugated secondary antibodies (Merck) for 1 h at room temperature, followed by detection using Pierce ECL reagents (Thermo Scientific, Rockford, IL, USA) and X-ray film exposure. The band intensity was semi-quantitatively analyzed using ImageJ software (NIH, Bethesda, MD, USA; version 2.0.0-rc-54/1.51h), with background values subtracted from the measured gray area of each band.

### 2.10. Cytokine Profiling

Thawed samples of circulating cell culture media were analyzed using the Human Cytokine Magnetic 10-Plex Panel (Invitrogen, ThermoFisher Scientific, Waltham, MA, USA), following the manufacturer’s protocol. This assay enables the quantitative measurement of GM-CSF, IFN-γ, IL-1β, IL-2, IL-4, IL-5, IL-6, IL-8, IL-10, and TNF-α. The Luminex™ platform utilizes microspheres labeled with distinct ratios of two fluorophores and conjugated to monoclonal antibodies specific for each cytokine. During the assay, the target cytokines bind to their respective beads, after which a secondary detection antibody is introduced. The beads are subsequently analyzed using a Luminex™ 100™ system. Standard curves were generated by adding serial dilutions of standards to the plate in duplicate. Samples were considered positive if their concentration exceeded the detection limits specified by the manufacturer.

### 2.11. Statistical Analysis

All experiments were conducted in triplicate, with the results expressed as the mean values ± standard deviation (SD). Before statistical evaluation, data normality was assessed using the Shapiro–Wilk test, which is appropriate for small sample sizes (<50). This test examines whether the data were consistent with a normal distribution; a *p*-value greater than 0.05 indicates that the data did not significantly deviate from normality, permitting the use of parametric statistical methods. As the data met the normality assumption, statistical analyses were performed using either a two-tailed independent Student’s t-test for comparisons between two groups or a one-way ANOVA followed by Dunnett’s multiple comparison test for analyses involving more than two groups. Statistical significance was defined as *p* < 0.05. All analyses were carried out using GraphPad Prism software (version 8.0 for Windows, GraphPad Software, San Diego, CA, USA), and the results are presented in the graphs with corresponding legends.

## 3. Results and Discussion

### 3.1. Fabrication of GDF-5 Releasing Nanocarriers

To induce the tenogenic phenotype, appropriate biochemical stimuli are required [10]. Growth major limitations Differentiation Factor-5 (GDF-5), a critical member of the Transforming Growth Factor-beta (TGF-β) superfamily, plays an essential role in tendon development and stem cell differentiation. However, the direct supplementation of growth factors in cell culture media is limited by their rapid degradation [29], which diminishes biological activity and requires repeated replacement during routine media changes. Furthermore, the high concentrations of growth factors required to achieve the desired biological effect (100 ng/mL in our case) [20] substantially increase culture costs [12]. To address this limitation, the first phase of the study focused on encapsulating GDF-5 within bioresorbable polymeric carriers, specifically Poly (Lactic-co-Glycolic Acid nanoparticles (PLGA-NPs), to enable the controlled and sustained release of the loaded peptide within a three-dimensional culture environment, thereby enhancing proper tenogenic commitment. GDF-5-loaded PLGA-NPs were fabricated by nanoprecipitation assisted by microfluidic technology, following a previously optimized protocol to ensure batch-to-batch reproducibility, precise particle size control, and suitable encapsulation efficiency [30]. Acetonitrile was selected due to its high-water solubility [31] and higher solvent power with respect to PLGA polymer, which facilitates more efficient mixing that leads to nanoprecipitation [32]. The staggered herringbone microfluidic chip employed in this study induces chaotic advection and transverse mixing within laminar flow conditions, significantly improving the mixing efficiency compared to conventional microchannels. This enhanced mixing facilitates rapid and homogeneous oil-to-water phases blending, resulting in improved control over the nanoparticle (NP) size distribution and uniformity [33,34].

### 3.2. Physicochemical Characterization of the Nanocarriers

**Figure 1:**
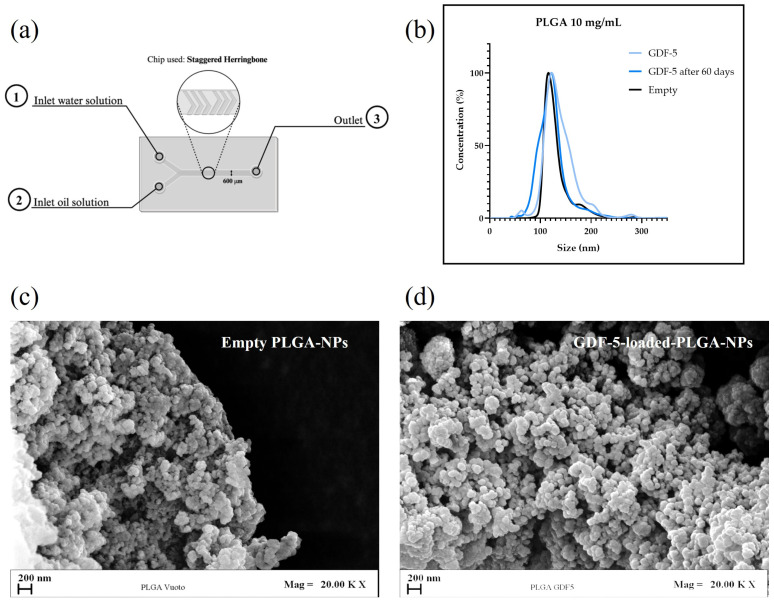
Characterization of GDF-5-loaded PLGA NPs fabricated by microfluidic assisted nanoprecipitation. (**a**) Schematic of nanoparticle (NP) fabrication, highlighting the inlets for the aqueous phase (1), oil phase (2), and the outlet (3). (**b**) Size-distribution curves of GDF-5–loaded NPs, loaded NPs after 60 days of storage to assess stability, and empty NPs used as a negative control. (**c**) SEM images of empty and (**d**) GDF-5–loaded NPs showing comparable morphology.

NPs were characterized by their particle size and ζ-potential, as summarized in Table 1. Field-Emission Scanning Electron Microscopy (FE-SEM) analysis (see Figure 1) revealed that the NPs exhibited a spherical morphology with smooth surfaces. The empty PLGA-NPs had an average hydrodynamic diameter of 130 ± 30 nm and a polydispersity index (PDI) of 0.11, indicating a monodisperse formulation (see Figure S1). Upon encapsulation of GDF-5, the particle size slightly increased to 140 ± 40 nm, with a PDI of 0.16.

The ζ-potential of the NPs was measured to assess their colloidal stability. Empty PLGA nanoparticles (NPs) exhibited a ζ-potential of −28 ± 0.32 mV. Following GDF-5 encapsulation, the ζ-potential increased slightly to −21 ± 0.58 mV. This shift may be attributed to the interaction between the protein terminal groups and the PLGA chains on the NPs surface, although the encapsulation process is expected to embed most of the protein within the polymeric matrix. Nonetheless, the observed reduction in ζ-potential is not expected to compromise NP stability in colloidal suspension and is consistent with the previous literature that reported similar values when small peptides were encapsulated [35]. After 60 days, the lyophilized material was resuspended and tested again for the ζ-potential: it still was negative, at −23.1 ± 1.15 mV, indicating the NPs’ stability over time, with proper storage. The stability observed over 60 days highlights the potential for long-term storage, facilitating its distribution and utilization. The encapsulation efficiency (EE) of GDF-5 within the PLGA nanoparticles was 33%, resulting in a final loading of 528 µg GDF-5 per gram of PLGA. This level of loading was considered adequate to sustain a tenogenic microenvironment following the incorporation of ~10 mg/mL NP into the 3D scaffold.

### 3.3. 3D Culture Establishment and Pathological hTSPCs Viability

All main plotting conditions are summarized in Table 2. The scaffold’s collagen concentration was maintained at 8 mg/mL (as already established [5]), and prior to transferring the final master mix into the syringe, PLGA-NPs were incorporated in the solution at 11.5 mg/mL. In this study, both GDF-5-loaded and empty NPs were employed; empty carriers were considered as a negative control for all the essays unless otherwise specified.

**Figure 2:**
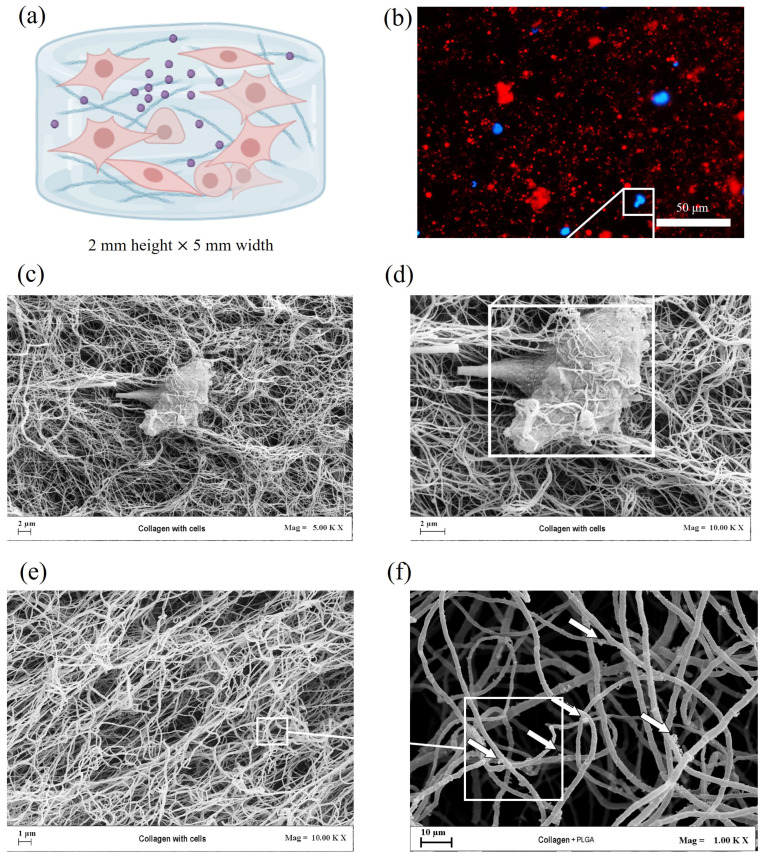
Characterization of 3D ColMA scaffolds. (**a**) Schematic representation of the cylindrical collagen-based scaffold with hTSPCs and PLGA-NPs. (**b**) Fluorescence microscopy image showing the distribution of rhodamine-encapsulated NPs (red) and cell nuclei (blue). Scale bar: 50 µm. (**c**–**e**) FE-SEM images of the collagen scaffold containing encapsulated cells at magnifications of 5000× (**c**) and 10,000× (**d**,**e**), illustrating cellular integration within the fibrillar collagen network. The white box highlights the correspondence between the DAPI staining and the cell in the FE-SEM micrograph. (**f**) FE-SEM micrograph at 1000× magnification showing the collagen fibers with NPs (white arrows), which appear distributed along the collagen fibers.

In more detail, after resuspending the collagen and neutralizing it, LAP was added at a final concentration of 0.25% *w*/*v*. Cells were then detached and resuspended in 250 μL of fresh cell culture media. Lyophilized NPs, both empty and GDF-5-loaded, were then resuspended in the same volume of the cells, in order to mix this solution with the collagen solution. The final master mix of neutralized collagen at 8 mg/mL, functionalized with LAP, cells, and NPs was bioplotted achieving a 3D scaffold as schematized in (Figure 2a). A confocal image of the scaffold was acquired, highlighting the NPs in red (by loading Rodamine B within the polymer) and cell nuclei with DAPI (see Figure 2b). From the image, it is clearly shown that the NPs were homogeneously distributed along the scaffold with cells. SEM micrograph analysis further confirmed the scaffold structure (Figure 2c,d). A prominent hTSPC is clearly identifiable within the fibrillar collagen matrix, demonstrating successful cell encapsulation and close integration within the scaffold architecture; conversely, the presence of NPs distributed along the scaffold filaments are also evident (see arrows in the zoomed area).

**Figure 3:**
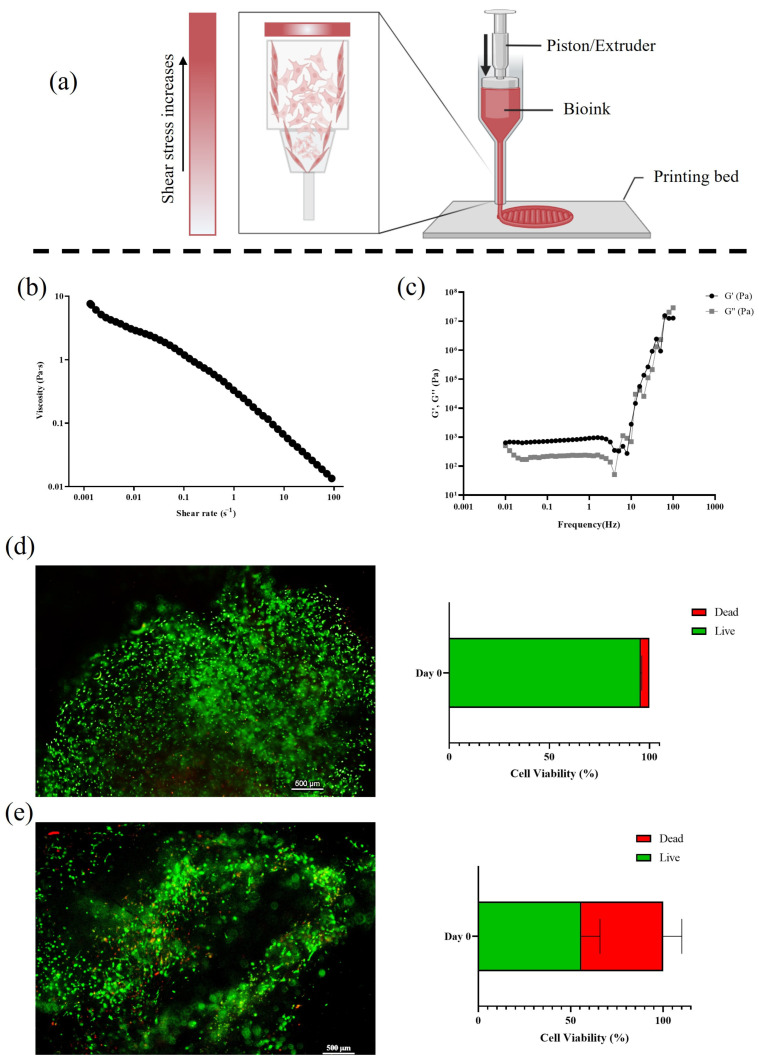
Shear stress impact during 3D bioprinting and evaluation of cell viability. (**a**) Schematic representation of shear stress effects on cell viability during bioprinting. (**b**) Viscosity profile of ColMA bioink as a function of the shear rate, illustrating its shear-thinning behavior, which is advantageous for cell printing. (**c**) Frequency-dependent storage (G′) and loss (G″) moduli of the 3D scaffold, characterizing its viscoelastic properties. (**d**) Live/dead staining micrograph and viability of hTSPCs within the ColMA 3D scaffold without NPs, at day 0. (**e**) Live/dead staining micrograph and viability within the ColMA 3D scaffold with 11 mg/mL of NPs (mean size 140 nm), revealing a reduction in viable cells. Scale bars: 500 µm.

In extrusion-based bioprinting, the shear stress from the nozzle extrusion can impact the cell survival by affecting the membrane integrity, cytoskeletal function, and overall cellular health [36]. Factors such as the nozzle diameter, extrusion pressure, bioink viscosity, and flow rate may influence the degree of the shear-induced stress. Additionally, UV crosslinking can further affect cell viability [37]. In this context, hydrogel thixotropic properties help mitigate the shear stress, as its viscosity decreases under mechanical stress during printing and recovers afterward, maintaining the scaffold structure and cell viability [38]. The process is schematically described in Figure 3a. The viscosity profile of the ColMA bioink and the related viscoelastic properties data are reported in Figure 3b,c. The ColMA bioink exhibited a pronounced shear-thinning behavior, as evidenced by the marked decrease in viscosity with the increasing shear rate observed in the flow curve analysis (Figure 3b). This rheological response is indicative of a non-Newtonian fluid, which is highly desirable for extrusion-based bioprinting processes [39]. The high viscosity at low shear rates contributes to the shape fidelity and mechanical stability post-printing, whereas the significant reduction in viscosity at higher shear rates facilitates smooth extrusion through the printer nozzle. These properties ensure that the scaffolds maintain both printability and structural integrity, supporting methacrylated collagen’s applicability in biofabrication contexts [40].

Crosslinked scaffold frequency sweep tests were performed in the oscillatory regime to determine the storage modulus (G′) and loss modulus (G″) as functions of the angular frequency (see Figure 3).

**Figure 4:**
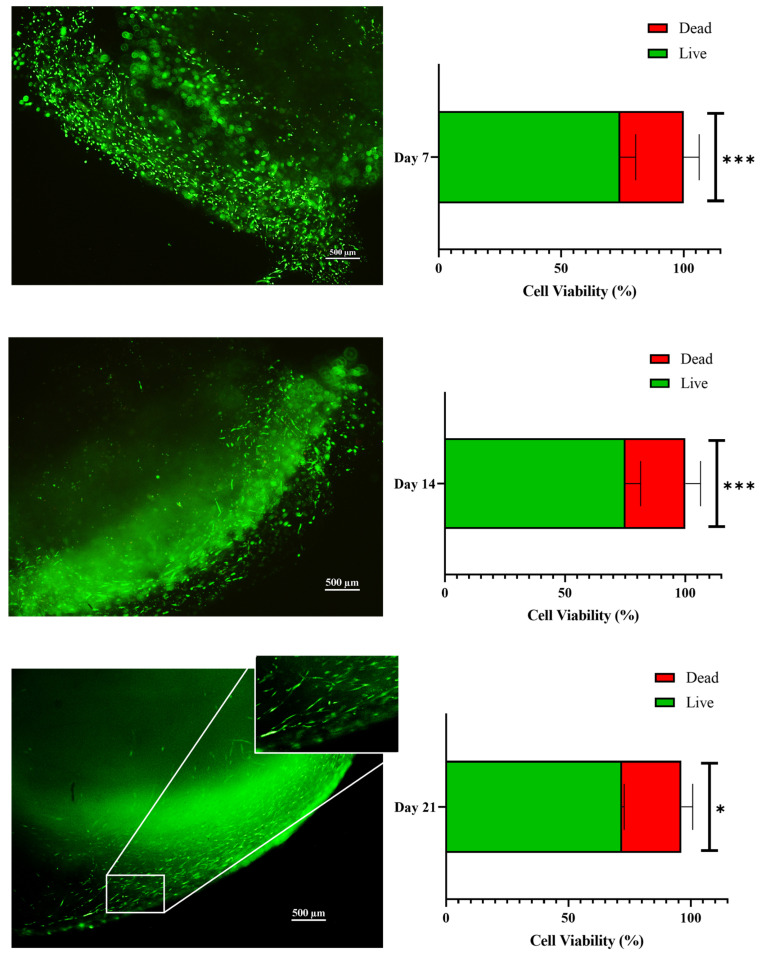
Live/dead assay results showing a progressive increase in cell viability of hTSPCs culture under perfusion (1 mL/min). Representative fluorescence micrographs (**left**) showing abundant live cells (green) at days 7, 14, and 21. Corresponding quantitative bar charts (**right**) indicate statistically significant increases in the percentage of live cells (*** *p* < 0.001 at days 7 and 14; * *p* < 0.05 at day 21), accompanied by a reduction in dead cells (red). Magnified views highlight uniform cell distribution and elongated cell morphology within the scaffold at later time points. Scale bars: 500 µm. Data are presented as mean ± standard deviation (N = 3 biological replicates).

The measurements were conducted at 37 °C using a parallel-plate geometry with a strain amplitude of 1% within the linear viscoelastic region. The predominance of G′ over G″ across the frequency range (0.01–100 Hz) indicates solid-like behavior and elastic dominance, essential for maintaining structural integrity during perfusion at flow rates such as 1 mL/min. This viscoelastic profile confirms the 3D scaffold’s suitability for perfused bioreactor applications, where mechanical stability under shear and oscillatory stresses simulates physiological loading in tendon tissue engineering. In addition, these properties ensure the scaffold withstands fluid flow without excessive dissipation of energy as heat, supporting long-term cell viability and extracellular matrix remodeling in tendinopathic models. Post-printing live/dead assays revealed an initial cell viability of 95% when pure ColMA was used loading 1 million of hTSPCs per mL (Figure 3d); these data are in agreement with those previously reported [5]. When NPs at a concentration of 11.5 mg/mL were added, a reduced cell viability of approximately 55% was observed (Figure 3e). This viability rate was notably lower compared to the control condition, in which hTSPCs were bioplotted in ColMa without NPs. The incorporation of NPs can influence the rheological behavior of the bioink and increase the shear stress during extrusion, potentially affecting the cell survival throughout the bioprinting process. Moreover, pathological primary cells are inherently difficult to manipulate because of their impaired phenotype and limited proliferative capacity. Therefore, the obtained post-printing viability can be considered a good outcome, particularly since comparable data on pathological tendon-derived cells were not found in the current literature. Despite their initially low viability, the cells adapted well to the 3D environment under dynamic perfusion. Quantitative analysis revealed a significant increase in viable cells at days 7, 14, and 21 with respect to day 0, accompanied by a significant reduction in dead cells over the same timeframe (Figure 4). Magnified views highlight uniform cell distribution and elongated cell morphology within the scaffold at later time points, suggesting proper differentiation. These results highlight the supportive effect of the 3D scaffold combined with the perfusion culture in sustaining long-term cell survival in vitro. Indeed, in our previous study, medium velocity was calculated both in the wells and through a similar 3D collagen scaffold and reported in about 3.42 × 10^−4^ m/s in the culture chamber and 2.95 × 10^−4^ m/s in the 3D environment, along the dynamic culture. These previously reported values suggest a good mass transport of metabolites through the 3D culture; at the same time, perfusion assured appropriate sink conditions near the NPs, enhancing the proper release rate of the biologically active load [5].

### 3.4. Sirius Red Collagen Staining and Difference Between Healthy and Pathological Tissue

**Figure 5:**
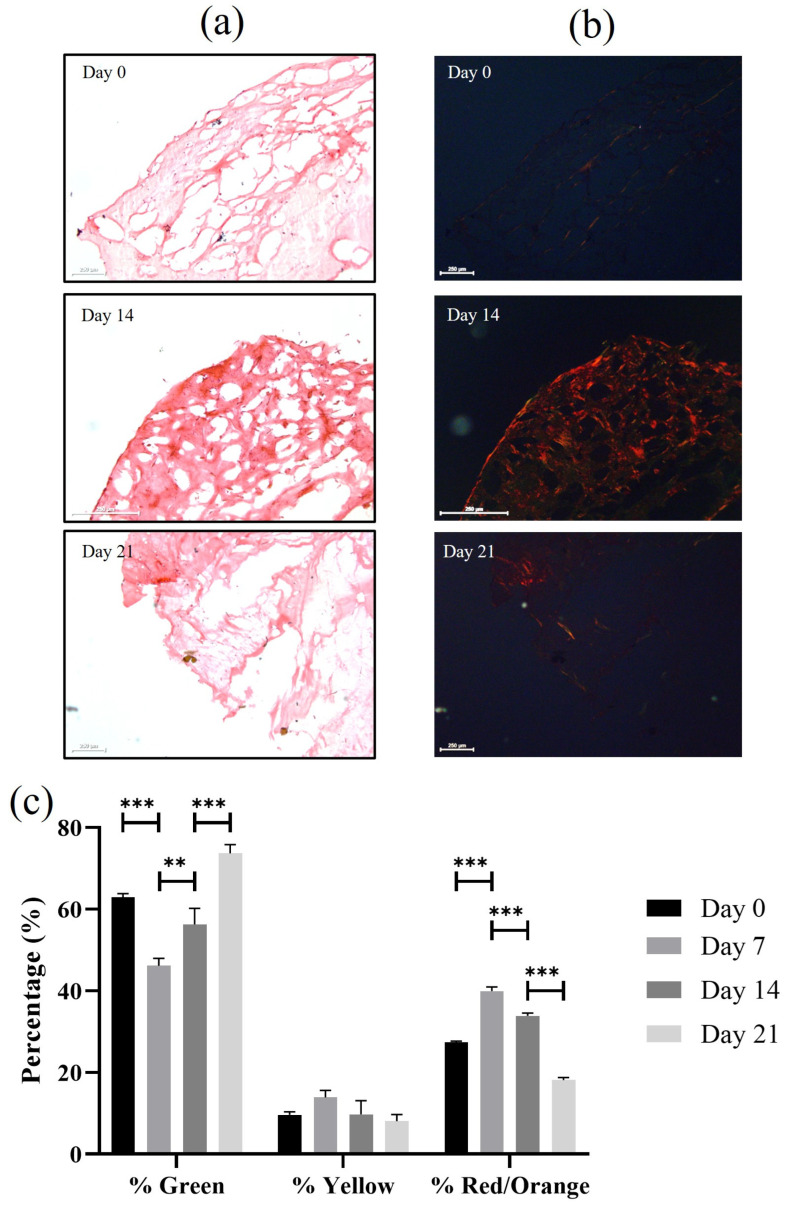
Picrosirius Red-stained sections of 3D scaffolds seeded with pathological hTSPCs cultured for 21 days. (**a**) Brightfield images at days 0, 14, and 21 showing increasing extracellular matrix (ECM) deposition. (**b**) Corresponding polarized light micrographs displaying predominantly green/yellow birefringence, characteristic of immature type III collagen, and minimal red/orange signals associated with mature type I collagen, most noticeable at day 14. (**c**) Quantification of birefringence color fractions, showing mean area percentages (±SD) of green (type III), yellow (immature type III), and red/orange (type I) signals across time points (N = 3). Green remains the dominant fraction, yellow increases modestly, and red/orange decreases significantly over time (*p* < 0.01), indicating fibrotic ECM remodeling and impaired type I collagen maturation in the tendinopathic model. Scale bars: 250 μm. *** *p* = 0.005, ** *p* = 0.05.

Sirius Red staining, analyzed under both polarized (Figure 5b) and brightfield microscopy (Figure 5a), provided a detailed assessment of collagen fiber deposition and organization by pathological cells over the 21 days of culture. Under polarized light, the type III collagen fraction, summed as green + yellow signals (Figure 5c), exhibited a baseline dominance of 72.59 ± 0.28% at day 0, reflecting the inherent fibrotic composition of tendinopathic explants upon initial seeding. A transient decline to 60.03 ± 0.98% occurred by day 7, potentially linked to early proliferative responses or scaffold integration. Subsequently, the type III fraction rebounded to 66.07 ± 0.64% at day 14, coinciding with the qualitative prominence of type III fibers noted in Figure 5, and culminated at 81.80 ± 0.56% by day 21, indicating progressive fibrotic accumulation and failure to transition toward mature matrix deposition. On the other hand, the type I fraction (red/orange) started at 27.41 ± 0.28% on day 0, peaked modestly at 39.97 ± 0.98% on day 7 (suggesting limited initial maturation), and then declined to 33.93 ± 0.64% on day 14 and 18.20 ± 0.56% on day 21, underscoring a persistent deficit in organized load-bearing collagen synthesis typical of impaired tenogenic differentiation in tendinopathy models.

This trend is indicative of poor fiber alignment and mechanical incompetence and integrates with the molecular evidence of sustained inflammation and reduced tenomodulin expression in the pathological hTSPCs. Such quantitative insights reinforce the platform’s utility for modeling tendinopathic ECM defects, where elevated type III persists despite culture duration, potentially due to altered mechanotransduction or cytokine signaling in the 3D microenvironment.

### 3.5. Tenogenic Gene and Protein Expression

Tenogenic differentiation and tendon tissue homeostasis are governed by the expression of specific extracellular matrix proteins and cellular markers that reflect both developmental and reparative processes. Key components of this regulatory network include type I and III collagen, tenomodulin, and tenascin-C, each playing distinct and complementary roles in tendon structure, maturation, and response to physiological or pathological stimuli [41,42,43]. Tenomodulin is a well-recognized marker of mature tenocytes and is closely associated with tendon development and maturation, reflecting tenogenic differentiation and extracellular matrix organization. On the contrary, type III collagen is a component particularly prominent during tissue remodeling and repair and is often upregulated in response to tendon injury or fibrotic changes [22,41,44]. Tenascin-C (TNC) is a large extracellular matrix glycoprotein that plays essential roles in tendon tissue homeostasis, remodeling, and response to mechanical stimuli. Within a tendon, TNC is typically found at sites subjected to high mechanical stress, such as the myotendinous and osteotendinous junctions, and its expression is highly inducible during embryonic development, tissue injury, inflammation, and regeneration [45,46].

**Figure 6:**
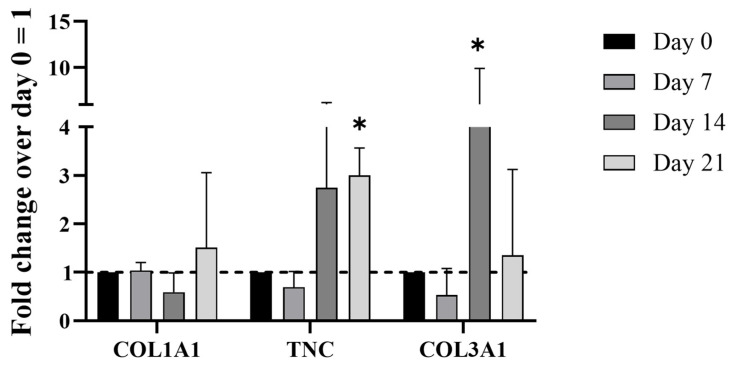
Gene expression profiling of key tenogenic markers of hTSPCs culture within the 3D scaffold. The chart displays the relative mRNA expression levels of *COL1A1*, *TNC*, and *COL3A1* at days 0, 7, 14, and 21, calculated as a fold change over the expression at day 0. The results highlight a significant upregulation of TNC and COL3A1 at day 14, while COL1A1 expression shows only minor changes. This expression profile suggests an impaired extracellular matrix remodeling process, with the overexpression of *COL3A1* being indicative of a fibrotic response often associated with chronic tendon pathology. N = 3 (biological replicates). * *p* = 0.05.

The qRT-PCR analysis is displayed in Figure 6, and it reveals the distinct temporal regulation of key tenogenic markers. The measured fold changes for *COL1A1*, *TNC*, and *COL3A1* normalized to day 0 indicate a generally modest induction of *COL1A1* across the culture duration, with only minor elevations at day 21. In contrast, *TNC* expression exhibited a notable increase, peaking around day 14, whereas *COL3A1* showed an even more pronounced upregulation at day 14, followed by persistent expression at day 21.

These findings are consistent with a previous paper showing that pathological tendon cells display impaired type III and type I collagen ratio expression, compared to healthy controls. The overexpression of *COL3A1* is often associated with fibrotic tissue assembly and pro-inflammatory environment. This altered expression profile underscores the dysfunctional regenerative capacity of pathological cells, with reduced organization of collagen fibrils as well as ongoing fibrotic processes, consistent with chronic pathology. Tenomodulin gene expression was not detected. The gene expression profile suggested an attempt at tenogenic commitment, albeit hindered by the pathological nature of the cells. The absence of this pattern in control experiments with empty PLGA-NPs supports the conclusion that GDF-5 was released in a sustained manner within the scaffold environment (data not shown). This finding supports the idea that the described modular 3D microenvironment, incorporating a GDF-5 reservoir, offers a promising platform for monitoring pathological processes.

**Figure 7:**
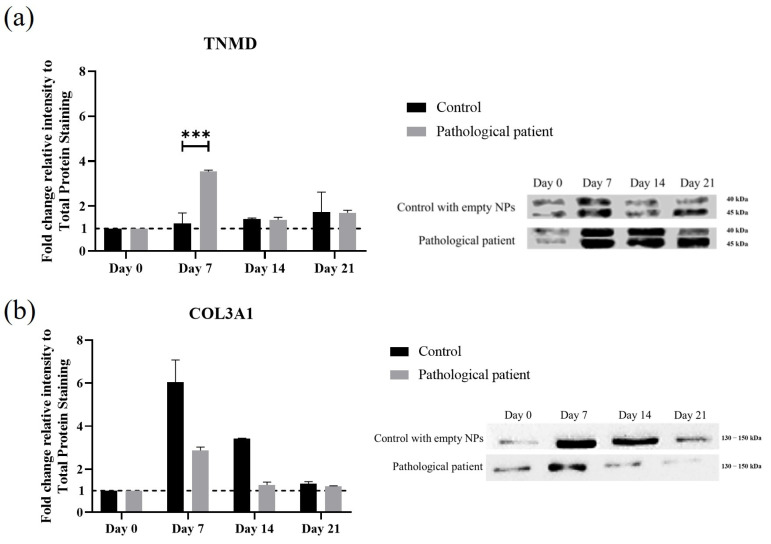
Protein expression profiling of key tenogenic matrix markers in pathological hTSPCs culture within a 3D scaffold system. Western blot quantification of TNMD (**a**) and COL3A1 (**b**) protein levels, normalized to total protein staining and comparing control and pathological patient-derived cells over time, with representative immunoblots illustrating marker expression dynamics. N = 3 (biological replicates). *** *p* = 0.005.

Further investigation of the ECM was performed by Western blotting. This molecular analysis allowed for the quantification of protein levels associated with tenogenesis and revealed distinct patterns of tenomodulin and collagen type I/III proteins during the 21-day culture period (Figure 7a,b). On day 7, tenomodulin appeared and type III collagen was elevated; type I was not detected. These findings underscore the divergent regenerative capacities of pathological cells with respect to that reported in the literature for healthy ones [43]. Finally, the dysregulated type III/I collagen ratio highlights compromised matrix deposition, which may contribute to deficient tissue remodeling and impaired recovery [47].

### 3.6. Cytokine Analysis and Quantification in Circulating Culture Media

**Figure 8:**
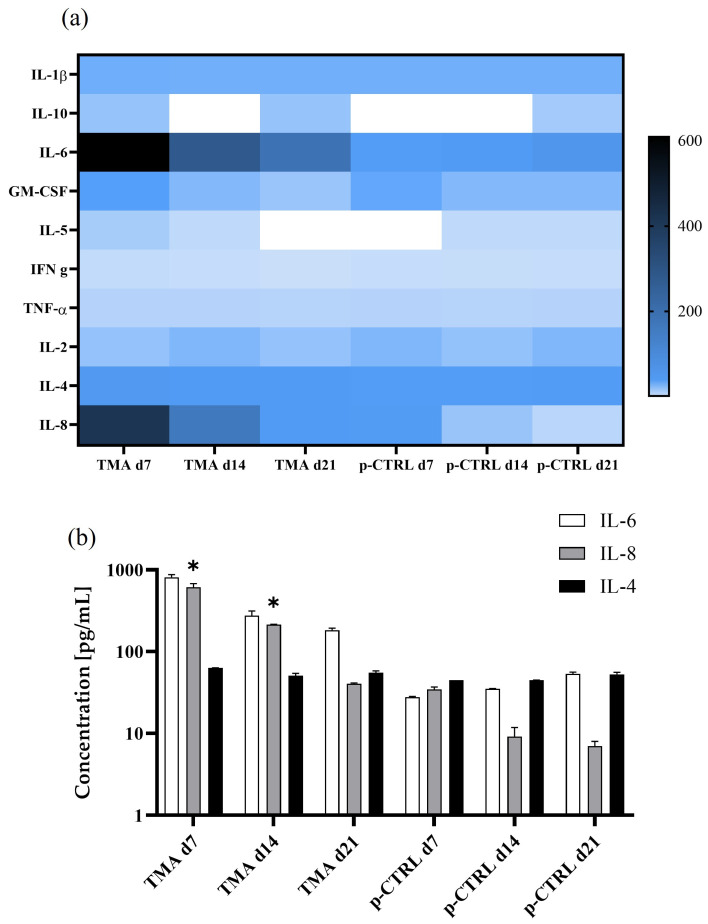
Cytokine secretion profile of pathological hTSPCs culture within a 3D scaffold system over 21 days. (**a**) Heatmap depicting concentrations of key cytokines (pg/mL) produced by TSPCs from tendinopathic matrix architecture (TMA) and pathological control culture (p-CTRL) over 7, 14, and 21 days. (**b**) Bar graphs quantifying IL-4, IL-6, and IL-8 levels at each time point for both groups. Data illustrate dynamic changes in pro- and anti-inflammatory cytokine production, highlighting distinct immunomodulatory responses in TMA versus p-CTRL conditions over time. Error bars indicate standard deviation from N = 3 biological replicates. * *p* = 0.05.

Cytokines are critical signaling molecules that regulate the complex interplay between inflammation and tissue regeneration during tendon healing [48]. The selection of cytokines analyzed in this study aimed to capture both pro- and anti-inflammatory pathways that are essential for understanding the immunomodulatory dynamics of tendon repair processes. Interleukin 4 (IL-4) acts primarily as an anti-inflammatory cytokine, modulating immune responses and limiting excessive tissue inflammation in tendon repair. IL-6 is a key pro-inflammatory mediator that drives cellular recruitment and matrix remodeling and can contribute to both regeneration and chronic tendon degeneration. IL-8 orchestrates early inflammatory responses and influences tissue remodeling during tendon injury and healing [48,49]. The quantitative analysis (Figure 8a,b) of these key cytokines demonstrated that the tendinopathic samples exhibited an extensive and persistent pro-inflammatory phenotype, especially during the early stages of culture.

In pathological (TMA) samples, IL-6 and IL-8 showed a rapid initial secretion with values of 763.98 pg/mL and 566.58 pg/mL at day 7, which then decreased to 190.03 pg/mL and 41.09 pg/mL by day 21, respectively. Comparatively, the control samples displayed substantially lower levels of IL-6 (ranging from 28.12 to 55.16 pg/mL) and IL-8 (ranging from 36.10 to 6.29 pg/mL) during the same period. Notably, both the heatmap visualization and the raw values underscore the pronounced upregulation of IL-6 and IL-8 as critical features distinguishing tendinopathic matrix environments from healthy or control conditions (with empty NPs). The observed dysregulation of pro-inflammatory cytokines in pathological cells aligns with current perspectives on the failed resolution of inflammation in tendon disease, as highlighted by Heinemeier et al. [50]. The authors reported that unresolved inflammation driven by cytokine imbalances, including elevated IL-1β and TNF-α, and persistent pro-inflammatory macrophage presence contributes to matrix disorganization, impaired differentiation, and fibrosis, as recapitulated in our in vitro model. The pathological cells’ overexpression of type III collagen (COL3A1), typical of scar tissue, further supports this concept. These findings resonate with the pro-inflammatory cytokine overexpression documented herein and the impaired maturation of pathological cells. Comparative analysis across these key studies confirms that the current 3D tendon pathological model captures critical aspects of cytokine imbalance, persistent inflammation, and matrix remodeling typical of tendinopathy, providing a reliable and translationally relevant tool for further investigation of the pathological conditions.

## 4. Conclusions

This study demonstrates that a 3D ColMA-based tenogenic microenvironment with GDF-5-loaded PLGA nanocarriers, under dynamic culture, provided an effective culture system to study the tenogenic events of pathological human primary stem cells. On the other hand, thanks to the NPs’ sustained delivery of differentiation growth factors, the 3D environment provided a bioactive synthetic ECM for observing cell behavior. Despite the NPs slightly affecting the cell viability along the plotting process, modifying probably the shear stress force, acceptable viability (exceeding 85%), was obtained for further investigation. The 3D synthetic ECM provided information about impaired ECM collagen balance with the progressive type III collagen fraction rising at day 21, while type I declined. The measured type III/I ratio of 4.5 reflects sustained fibrosis, likely resulting from an overall pro-inflammatory cytokine environment. Functionally, the 3D environment facilitated the observation of both cellular behaviors and matrix deposition patterns relevant to study tendon pathology. The inflammatory signature correlated closely with disruptions in ECM organization and stagnation of tenogenic protein accumulation, underscoring the pathological inability of the cells to achieve a successful regenerative response.

These findings provided new insights into the role of biomaterial-based scaffolds and growth factor delivery in modulating stem cell behavior and may contribute to further study of specific treatment for chronic tendinopathies. The study offered also insights into the groundwork for personalizing regenerative treatments for tendon injuries, supporting the development of next-generation therapeutic solutions tailored to individual patient needs.

## Figures and Tables

**Table 1:** Summary of the PLGA-NPs main physicochemical parameters investigated, related to PLGA nanoparticles.

Sample: PLGA	Mean Size (nm)	PDI	ζ-Potential (mV)	Loading $\left(\frac{µg}{g}\right)$
Empty	130 ± 30	0.11	−28 ± 0.3	--
hGDF-5	140 ± 40	0.16	−21 ± 0.6	528
hGDF-5 60 days	123 ± 30	0.26	−23 ± 1	--

**Table 2:** Summary of the main bioplotting conditions used during the extrusion process.

Parameter	Value	Notes
Print Speed	10 mm/s	procedure effectively ended in few minutes
Nozzle Diameter	22 G	precision and excellent cell viability
Printing Temperature	18 °C	low T to be properly extruded
Bed Temperature	40 °C	start thermal crosslinking
Post-processing	UV plus 37 °C	required

## Data Availability

The original contributions presented in the study are included in the article/Supplementary Material, further inquiries can be directed to the corresponding author.

## Supplementary Materials

The following supporting information can be downloaded at: https://www.mdpi.com/article/10.3390/bioengineering12121337/s1, Figure S1: Dynamic Light Scattering Analysis of PLGA Nanoparticles.
