# Supplementary material for: A 3D ColMA-Based Tenogenic Microenvironment Unveils the Behavior of Tendon Stem/Progenitor Cells (TSPCs) from Tendinopathic Surgical Explants
Source: Bioengineering (Basel). 2025 Dec 8;12(12):1337. doi: 10.3390/bioengineering12121337 (PMC12729981; doi:10.3390/bioengineering12121337)
Supplement: Supplementary file 1 [file bioengineering-12-01337-s001.zip › bioengineering-3974513-supplementary.pdf]

Supplementary Materials:

Dynamic Light Scattering (DLS) analysis was performed to characterize the physicochemical properties of both empty and GDF-5-loaded PLGA nanoparticles prepared by microfluidics-assisted nanoprecipitation. ζ-potential measurements were used to assess colloidal stability and surface charge modifications associated with GDF-5 encapsulation, while intensity-weighted size distributions provided information on particle homogeneity and the impact of bioactive molecule loading. Samples were analyzed at a polymer concentration of 10 mg/mL, and both formulations displayed narrow and monodisperse size distributions, consistent with efficient nanoprecipitation and the stabilizing effect of the microfluidic process. The slight increase in average particle size and the shift in ζ-potential observed upon GDF-5 loading further confirmed successful encapsulation and modification of nanoparticle surface characteristics. These results validate the robustness and reproducibility of the platform for producing homogeneous and functionalized nanocarriers for controlled bioactive delivery in tissue-engineering applications.

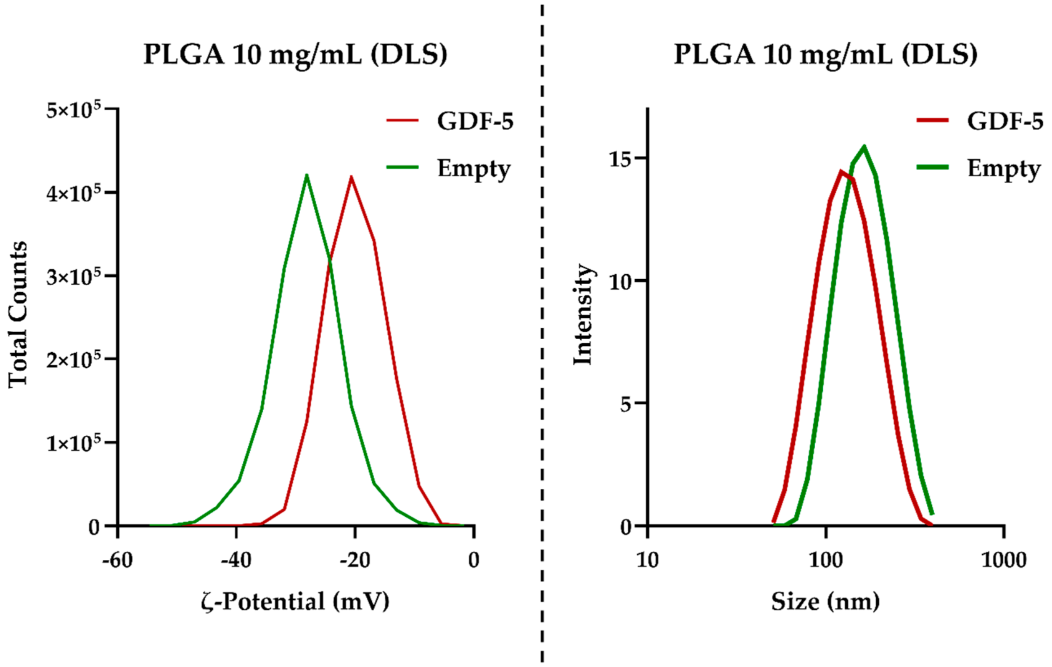

Figure S1. Dynamic Light Scattering Analysis of PLGA Nanoparticles.
(Left) Histogram of ζ-potential (mV) distribution showing total counts for empty (green) and GDF-5-loaded (red) PLGA nanoparticles. (Right) Particle size distribution represented by intensity versus diameter (nm) for both formulations. DLS analysis demonstrates that the inclusion of GDF-5 results in a slight shift in ζ-potential and particle diameter compared to empty nanoparticles, indicating successful encapsulation and surface modification. Data are representative of typical batch preparations.
